# Challenges of Trachoma Control: An Assessment of the Situation in Northern Nigeria

**DOI:** 10.4103/0974-9233.80699

**Published:** 2011

**Authors:** Mansur M. Rabiu, Nasiru Muhammed, Sunday Isiyaku

**Affiliations:** Director of Programmes, Prevention of Blindness Union, Riyadh, Saudi Arabia; 1 Ministry of Health Birnin Kebbi, Kebbi State, Nigeria; 2 Sight Savers International, Nigeria office Kaduna, Nigeria

**Keywords:** Control, Nigeria, SAFE, Trachoma

## Abstract

Over the last three decades, a lot has been achieved in the control of trachoma worldwide. New assessment techniques, effective evidence-based control strategy with new methods and drugs, and an aggressive global partnership for the control of the disease have evolved. As such the number of people with the disease and blindness due to the disease had drastically reduced. Trachoma is now only responsible for about 4% of blindness worldwide down from 12% some few decades ago. Some countries are on the verge of eliminating the disease as a public health problem. Despite these achievements numerous challenges remain for achieving trachoma control in endemic communities. This article highlights the challenges faced in one of the known trachoma endemic areas – northern Nigeria. Aspects on the dearth of complete situational data on trachoma, fragmented implementation of the SAFE strategy, community apathy, difficulties faced in ensuring safe, and quality lid surgery in the most difficult terrain where the disease thrives are discussed here. Other unique challenges like managing children with severe trichiasis, curbing the high rate of early-onset recurrence of trichiasis after lid rotation surgery and challenges to maintain supply of antibiotics and implementation of facial cleanliness and environmental improvement components of the control strategy are presented along with the learnt experiences and recommendations. These challenges and their remedies are likely to be shared by other trachoma endemic areas in Africa.

## INTRODUCTION

Trachoma is an infective condition of the eyelid that results in in-turned eyelashes, thus damaging the cornea and subsequently leading to blindness. In the 80s, trachoma was one of the major causes of blindness worldwide, when it constituted about 12% of world blindness.^1^ The distribution of trachoma corresponded with that of poverty in much of Africa and Asia. The risk for the disease was defined by conditions that facilitate transmission of the infecting organism—*Chlamydia trachomatis*—such as household crowding and limited access to and use of water.^2^ The World Health Organization (WHO) recommended trachoma control SAFE strategy comprising four components: (1) Surgery to correct trichiasis, (2) Antibiotics to treat active disease; (3) Facial cleanliness to reduce transmission; and (4) Environmental improvement to control determinants of transmission. By attending to the medical, behavioral, and environmental determinants of trachoma, the SAFE strategy addresses the immediate risk of blindness as well as root cause of the disease.^3^ This progress provided the basis for the World Health Assembly^4^ to call for the elimination of blinding trachoma by the year 2020, which was underscored by an additional resolution.^5^

The concerted efforts by stakeholders including WHO, Non-Governmental Organizations (NGOs), governments, and other agencies, coupled possibly by improvement in general socioeconomic conditions, has resulted in a decline in the incidence of the disease. The latest WHO estimates report that trachoma is only responsible for about 3% of blindness. There are currently estimated to be 40.6 million people with active trachoma, which has decreased from an estimated 84 million less than a decade ago. Despite this progress, the physical and economic burden of trachoma is even higher than previously projected. An economic assessment had asserted that trichiasis in itself may result in an economic burden comparable to trachomatous low vision and blindness.^6^

Every year, a critical meeting of the World Health Organization, the Alliance for the Global Elimination of Trachoma by 2020 (GET2020), is held to chart the progress toward this goal. Country representatives and their partners report on national programs, progress and/or constraints encountered, and plan for the coming year. Success is being reported from Morocco, Ghana, and Mexico, and likely success will soon follow in Vietnam, Oman, and others.^7^

**Figure 1:**
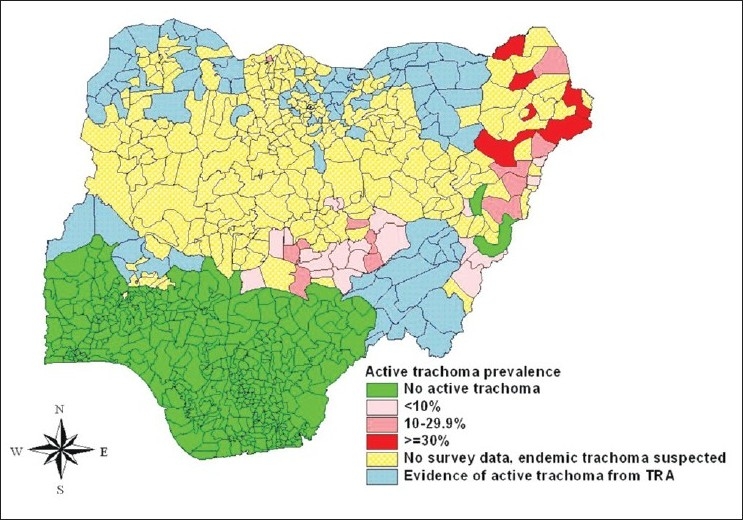
Distribution of active trachoma in children <10 years in Nigeria (as up 2006)

**Figure 2:**
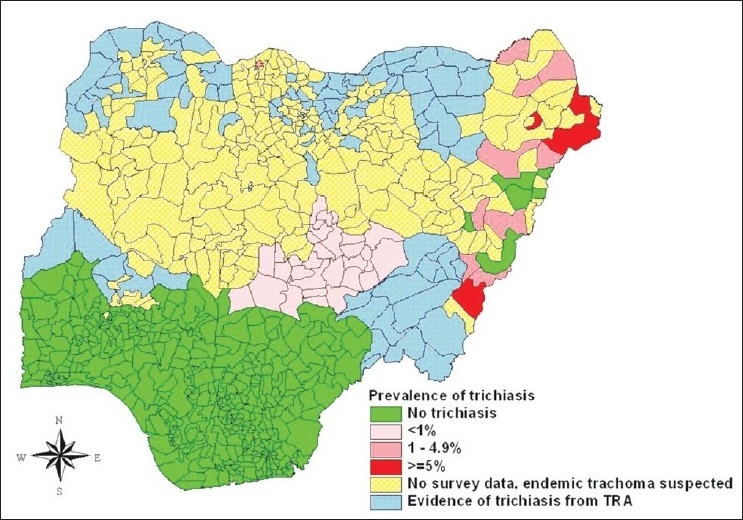
Distribution of trichiasis in adults >15 years in Nigeria (as up 2006)

Until 1999 when a population-based survey reported trachoma of public health significance in parts of northern Nigeria,^8^ previous reports had suggested that trachoma was not a public health problem in the country. These initial reports were mainly from hospital-based studies.^9^ More population-based surveys across several states of northern Nigeria later reaffirmed that trachoma of public health concern indeed exist in Nigeria.^10^–^12^ The prevalence of the disease is in the range 0.6%-17.6% for Trichiasis and 5%-49% for active trachoma across the trachoma belt of Nigeria. Even though a complete mapping of trachoma in northern Nigeria has not been done, several disease foci across the region have been identified [Figures 1 and 2]. Various NGOs and local governments are now involved with trachoma control in northern Nigeria. These include Sightsavers, Christoffel Blindenmission (CBM), The Carter Center, and Helen Keller International (HKI). These NGOs have formed a trachoma control forum where they share ideas and resources in the control of trachoma in various parts of the country. Currently, the health administration in Nigeria has placed trachoma control under the Neglected Tropical Diseases (NTDs) control program of the Federal Ministry of Health.

However, trachoma control in Nigeria is facing numerous challenges, many of which may be similar to other trachoma endemic areas in Africa.

## CHALLENGES

### Trachoma mapping and control programs

Many districts (local government areas) of northern Nigeria have some data on trachoma, which is based on either trachoma rapid assessment or an epidemiological survey. However, other districts of the region are yet to be mapped [Figures 1 and 2]. This is probably because the trachoma belt of northern Nigeria is vast. More than half of the 774 districts of Nigeria are in northern Nigeria, covering over 500,000 km^2^, with a population of over 80 million people. As such undertaking district by district population-based surveys to cover this vast area will require enormous resources and time. And NGOs as the main advocates and implementers of trachoma control in the country, are unable to provide such resources. A dearth of this comprehensive data had been a constraint for national trachoma control planning and resource utilization. Some states have a trachoma control program but most of them are not implementing the full SAFE strategy as recommended by WHO. Some states like Sokoto, Zamfara, and Kebbi have initiatives based mostly on the interest of the partnering NGO to provide community-based lid surgery only, while other states like Plateau and Nassarawa implement the personal and environmental hygiene components only. Local/Federal Governments and NGOs need to mobilize resources to complete the mapping of the entire trachoma belt of northern Nigeria. This can be integrated with the ongoing mapping of other NTDs. For optimal effect of the trachoma control program service providers, especially NGOs, should collaborate with other agencies and organizations to implement a full scale SAFE strategy.

### Community perspectives and participation in trachoma control

The ambivalent attitude of people to health services appear to be a universal phenomenon in many local communities in Africa, and perhaps hinges on the local people’s perspective of the disease, which varies from place to place. A study conducted in The Gambia concluded that for any intervention strategy to achieve the set goals of eliminating trachoma in spite of other constraints, community support and participation is essential, and in order to achieve this, the healthcare provider needs to have a better understanding of the community perspectives of the disease.^13^

Although we could not access any paper reporting community perspectives in Nigeria, it is observed that aspects of community participation are essential for optimal uptake of services like lid surgery and antibiotics usage. This was observed during a focused group discussion conducted in some districts with poor uptake of lid surgery.^14^ The discussion was aimed at determining the reasons for poor uptake of TT surgery following the finding of a low trichiasis surgical coverage (TSC) of 9.5%-12.5% from a population-based survey in districts where community-based surgery is provided free.^15^

The study identified stakeholders’ commitment to program implementation as the major challenge. There is a decline in stakeholders’ commitment to the program after 4 years of implementation. Problems identified included poor motivation (including per diem) and support to the primary healthcare workers (who are expected to do proactive case finding and referral); lack of mobility to the lid surgeons to reach communities for service delivery; poor communication between service providers and program management, and sometimes communal conflicts between neighboring communities. This demonstrates the importance of periodic review and studies to identify obstacles in program implementation, so as to address such problems.

### Lid Surgery and Antibiotic interventions

The WHO recommended strategy for the control of trachoma-SAFE is an effective control strategy but there are challenges to its application. Lid surgery and antibiotics distribution are two common intervention strategies used by trachoma control programs.

#### Community-based lid surgery

The lid surgery involves out-turning of the in-turned upper eyelid in a community setting. This intervention is more commonly undertaken by NGOs and governments because it is easy to demonstrate its impact compared to other components of the intervention. However, even this intervention has many challenges. These include questions about who does the community-based lid surgery, how to sterilize surgical instruments in a community setting, how to ensure high quality of surgery in a community setting, and the universal issue of recurrences of trichiasis after lid surgery.

**Figure 3:**
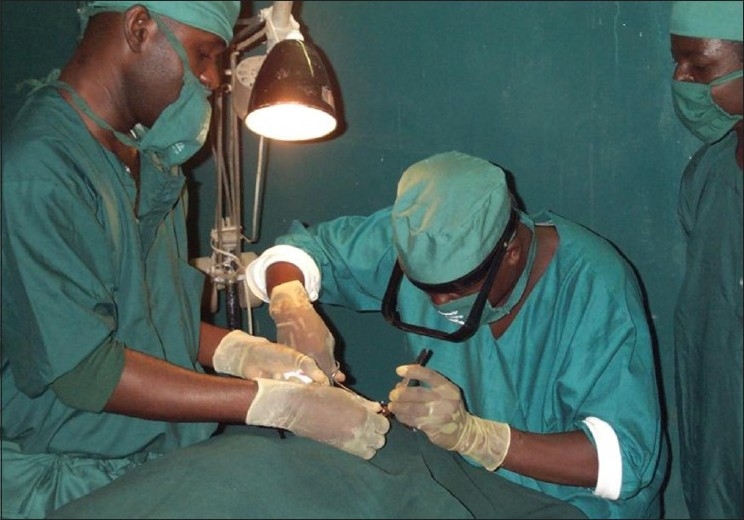
Nurses being trained as lid surgeons

**Figure 4:**
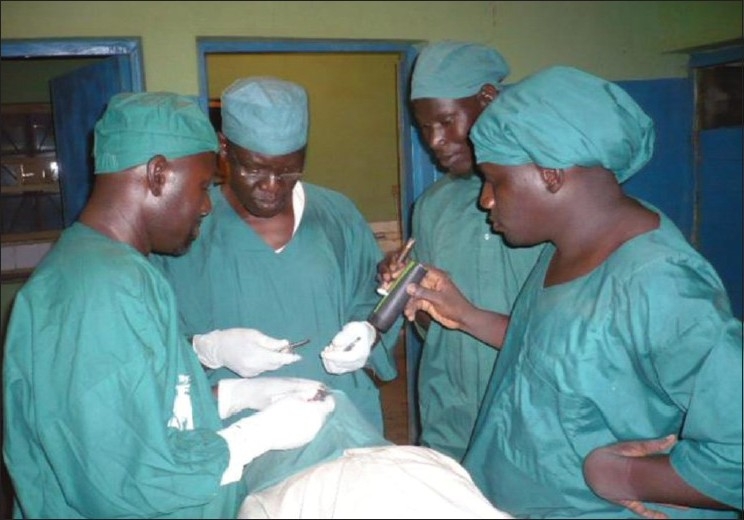
Nurses being trained as lid surgeons at a PHC center

In most trachoma endemic areas, there is a dearth of eye care personnel. Often, it is difficult to identify the available cadre to appropriately provide lid surgery. In northern Nigeria with limited number of ophthalmologists to be able to provide lid surgery services at community levels, ophthalmic nurses have been trained to do lid rotation surgeries based on the available evidence elsewhere that a non-ophthalmologist can perform the surgical procedure satisfactorily [Figures 3 and 4]. In Ethiopia and Morocco, such trained nurses had similar outcome as ophthalmologists.^16^–^18^ However, training nurses to do the lid surgery have met resistance in some states of Nigeria because some doctors feel that nurses are legally not allowed to operate on eyes. Such professional conflicts need to be resolved by the health administration in conjunction with professional bodies and their licensing organizations. Depending on the need in the areas and the available eye care personnel, the health and eye care practitioners should be able to work out an amicable position that will not deny the needy trichiasis patients the services they deserve.

The issue of maintaining the sterility of operating instruments is a big challenge, especially in this age of HIV pandemic and other blood borne transmissible diseases. An evaluation of the trachoma control program in two states of north-west Nigeria in 2008^16^ expressed concern on the level of sterility of operating equipment in some service centers. It was noted that some lid surgeons do not adequately sterilize their instruments in between surgeries either because sterilization takes long to take effect using an oven or they have inadequate number of instruments to replace used ones undergoing sterilization. The provision of large number of instruments sets to allow for effective sterilization of used instruments, as well as adoption of simpler and fast adjunct effective sterilization techniques like chemical sterilization methods needs to be identified. Furthermore, surgeons need to be regularly retrained on the importance and procedures for aseptic measures.

#### Recurrence of trichiasis

**Figure 5:**
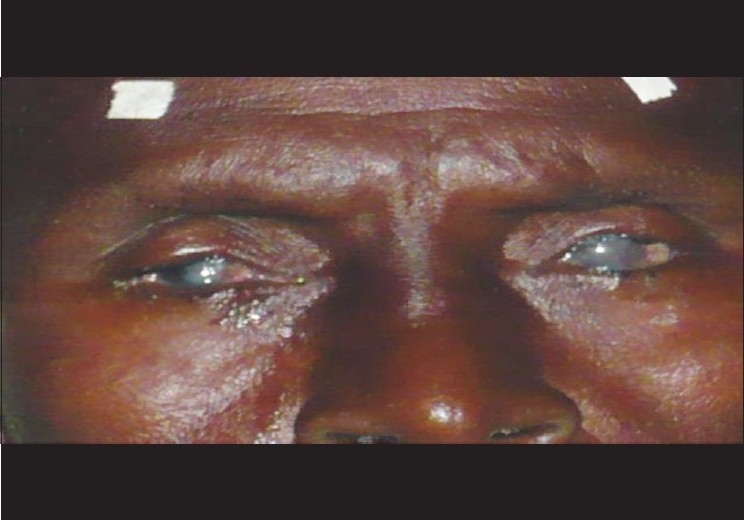
An adult with recurrent trichiasis

Recurrence of trichiasis after lid rotation surgery has been reported in various parts of the world^18^–^22^ [Figure 5]. A number of factors have been implicated, including poor surgical techniques, intense recurrent infections, healing responses, and long duration since the surgery.^17^ In a trachoma control program in northern Nigeria, more cases of early-onset recurrence (recurrence occurring within 3 months of surgery) were noted among some surgeons. The surgical skills of the surgeons were noted to have depreciated, with poor attention to surgical details like forceps placement on lid, incision site/size and suture placement, thus resulting in inferior quality surgeries and a high incidence of the early-onset recurrence. In one district, almost half of the cases done were either not done properly or had recently reoccurred. This was ascribed to a surgeon who was trained 4 years ago with no refresher course. A refresher course was subsequently organized by an NGO to address the depreciating skills, after which the quality of surgery improved with good lid rotation and less early-onset recurrence. In Ethiopia, surgeon, and incision size were reported as some of the causes of early-onset complications of trichiasis surgery, including trichiasis recurrence. The study reported that eyelids with short incisions were nearly four times more likely to have recurrent trichiasis (95% CI: 1.7-9.3)^18^ Regular refresher courses, supervision of surgeons, and self-auditing techniques not only boost the morale of surgeons but also assist them to pay attention to surgical details over time. Thus, interval refresher courses and regular supervisions are necessary for maintenance of good surgical outcome.

In view of plausible linkage of postoperative recurrence of trichiasis with recurrent infections or intensity of infections, the use of preoperative antibiotics has been advocated. However, the effect of such perioperative antibiotics to reduce re-occurrence has not been proven. In fact, a study in a low trachoma intensity area of the Gambia showed no difference in the recurrence between patients treated with azithromycin immediately after surgery and those not treated.^19^ However, a separate study in Ethiopia had demonstrated the efficacy of azithromycin in reducing recurrence compared to tetracycline ointment, such that patients receiving azithromycin had significantly fewer severe trichiasis recurrences, 4.2/100 person years overall, compared to those randomized to topical tetracycline, 7.9/100 person years (*P* < 0.01).^20^ More research in the form of randomized control trials in different settings across the world are needed to address this question.

#### Which cases of trichiasis to operate?

**Figure 6:**
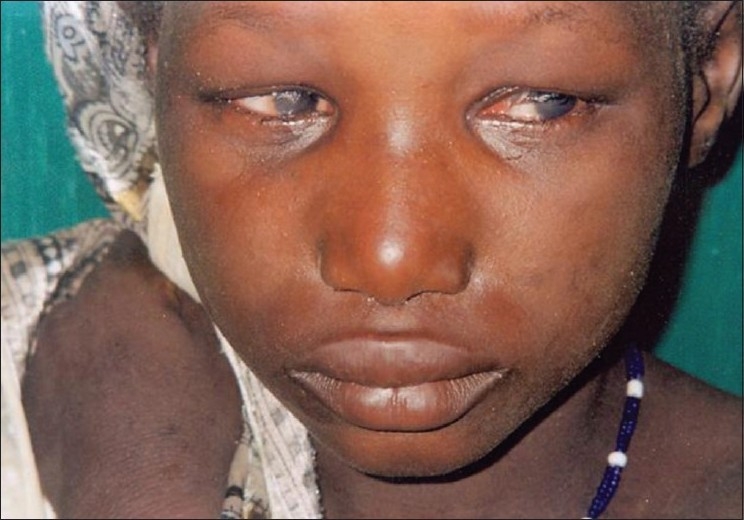
A 6-year-old girl with severe trichiasis and corneal opacities in northern Nigeria

**Figure 7:**
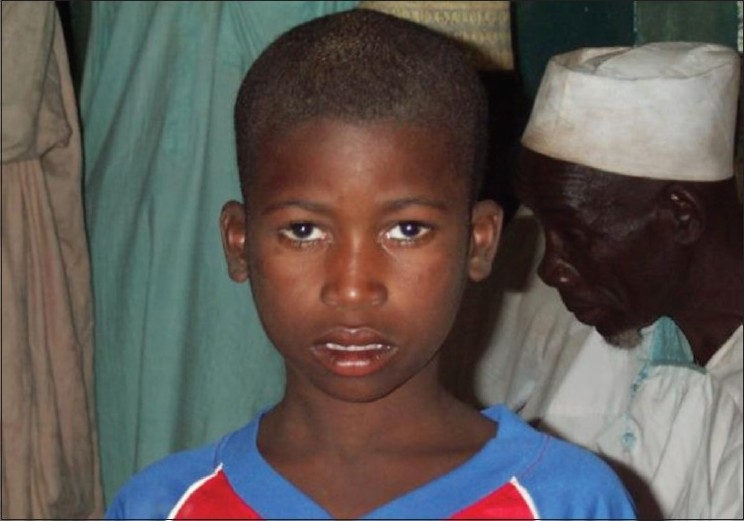
An 8-year-old boy with bilateral trichiasis

One of the difficulties faced by lid surgeons is the decision on which severity of trichiasis to operate on adults and the issue of children with trichiasis. Most people with one or two offending eyelashes are encountered in the field and different control programs have differing approaches to such mild trichiasis. Basically, important considerations in deciding the approach will include the backlog of severe trichiasis in the area, expertise of surgeons, effectiveness and efficiency of the control program to provide services later to such cases when the trichiasis becomes severe. In many control programs in Nigeria, mild cases are given palliative treatment in the form of epilation in order to concentrate on the many severe cases. Children as young as aged 6 years have been seen with severe trichiasis in some trachoma endemic communities of northern Nigeria [Figures 6 and 7]. This is an uncommon occurrence in other trachoma-prevalent areas of the world. This poses a unique challenge to the provision of lid surgery because children are unlikely to withstand this surgery under local anesthesia. Some surgeons had used dissociative anesthesia by using Ketamine for surgery. However, this method falls below normal safety requirements, and thus cannot be recommended. The trachoma control programs have had to refer children with trichiasis to the central hospital for surgery under general anesthesia. Thus, trachoma control programs have to be planned for such rare and complex situations of dealing with children having trichiasis.

#### Use of Antibiotics in control programs

Use of mass antibiotics distribution or targeted treatment is a major component of trachoma control program. Traditionally, tetracycline eye ointment has been used, but the use of azithromycin had been adopted by many programs because of its similar efficacy as the ointment and higher compliance, as it is an oral preparation given once in 6 months or per year. The major challenge to this control component is the cost of the drug. Even though the International Trachoma Initiative (ITI) in conjunction with Pfizer is donating azithromycin to some countries, the trachoma control programs in Nigeria have been unable to obtain the drugs, until recently, when approval was given for pilots in 17 districts in northern Nigeria in 2010. For now most NGOs involved in trachoma control in Nigeria use tetracycline eye ointments in few communities, as they are unable to provide the drug to all endemic communities. Although this effort is laudable, it has limited effect because of the expected cross-infections that can occur between neighboring villages. Furthermore, the oily nature of tetracycline and its daily usage for several weeks gives rise to limited compliance rate. One NGO provided azithromycin and used it in its control program in a district but had to abandon this approach due to high costs., The program could only be implemented in instances when ITI/Pfizer donation was obtained.

A multi-country evaluation also reported insufficient antibiotic distribution compared with the magnitude of the disease burden. In some countries, the trachoma control program covered only a small proportion of the population in need.^21^ It is recommended that azithromycin be made readily available to all endemic communities in the world. The recipient countries, their partners (NGOs), and communities must make adequate logistic and administrative plans to receive, distribute, and ensure effective use of the drugs and monitor utilization of the drugs. Early analysis of large-scale trachoma control programs in Africa suggests that the marginal cost of treatment delivery is similar to those reported by the African Program for Onchocerciasis Control and the Global Alliance to Eliminate Lymphatic Filariasis (<$0.50 per person). A more fundamental question revolving around is: who would financially support these programs when they strive to implement large scale intervention?^22^

### Facial Cleanliness and Environmental Hygiene interventions

A more sustainable and probably more effective control strategy for the trachoma control program is improvement in personal and environmental hygiene. However, it is more difficult to attain, as it takes time for the effect to be visible and requires attitudinal and often sociocultural changes. These parameters may take generations to change, especially in isolated communities with no modern social and educational facilities. The primary school enrollment in Nigeria is about 68% and 58% for boys and girls, respectively.^23^ In rural areas, this figures are much lower; in fact, it is close to zero in some communities. So perpetuation of old unhealthy habits is likely. For example, the common practice of defecating in open field or breeding animals in household compounds, both of which are prominent risk factors to proliferation of houseflies that transmit the disease, is unlikely to easily disappear. Indeed, in Nigeria, only about 30% of the populace has access to proper sanitary system, and in rural areas this percentage drops to less than 25%.^24^ This is even worse in the very remote areas where entire hamlet with no sanitary system is a common feature.

Furthermore, hygienic practices are closely related to use of water. In Nigeria, less than 50% of adequate water sources is available for people; and in rural areas where trachoma thrives, this is even less than 30%.^24^ Most communities of the trachoma belt in Nigeria are in arid dry areas, with severe scarcity of water, especially during some seasons of the year. Wells, which are the most common source of water, may be dry for half of the year. Rain water harvesting has been suggested in some regions of Africa where limited water is available. However, rain water in some communities is scanty in less than 3 months of the year, where average annual rainfall is less than 400 mm. Some regions of northern Nigeria adjoining Niger republic had recently been experiencing droughts due to low rainfall, which worsens the already bad situation. Thus, the limited harvested water cannot be used the year round. In some terrains, the water table is very low and requires very deep wells, and thus the cost of reaching the water is high. The deep wells and boreholes constructed in some communities by local authorities only work for few months or weeks and then become dysfunctional. The persistent scarcity of drinking water, not to mention water for sanitary purposes, means that proper use of water for personal and environmental purposes is often seriously limited.

Addressing the issue of water scarcity and sanitary conditions in vulnerable communities requires higher investment, which neither the governments nor NGOs are often ready to undertake. The difficulty in undertaking these projects is compounded by the fact that it is not solely a health issue but one that needs multi-sectoral approach linking ministries and agencies concerning water, environment, education, women affairs, etc. Awareness about trachoma, the SAFE strategy and use of water should be promoted. The government and NGOs should encourage such activities and build local capacity for the management of water and sanitation resources in affected areas. Each country needs to adapt the elements of the SAFE strategy to its own unique conditions.^25^

Many NGOs may not be willing to address the problem because it is capital intensive and does not show results immediately, as the impact manifests many years after. Furthermore, there is limited clear scientific evidence that FE intervention really works to prevent trachoma. Although a study reported disappearance of trachoma in a village without a control program, this was attributed to improvements in sanitation, water supply, education, and access to healthcare in the village.^26^ A recent report from Ethiopia suggests that simultaneous implementation of ‘AFE’ components is associated with less odds of active trachoma in children.^27^ However, comprehensive systematic reviews undertaken had reported differing end results. A Cochrane systematic review on face washing intervention and another on environmental sanitary interventions, concluded that more evidence is required to confirm the efficacy of these interventions to control trachoma.^28^,^29^

All the same, some NGOs like The Carter Center and the Sightsavers have been involved in the FE control of trachoma in Nigeria, where they have been encouraging building of Ventilated Improved Pit (VIP) latrines in communities through limited cost of materials and labor. The objective is to enhance personal and environmental hygienic practices through multi-focal health education activities and provision of sanitary ware to endemic communities. Sanitary wares (Diggers, rakes, head pans, shovels, wheel barrows) were provided to 30 communities in one of the Sightsavers supported projects. These items are being used by communities to keep the environment clean, thereby improving environmental hygiene. Water and sanitation committees have been formed in each of these 30 communities to encourage, monitor, and promote environmental cleanliness in communities. Masons were also trained in 30 communities on construction of pit latrines. They also worked with local government staff to construct demonstration latrines.

Advocacy to local government and communities for involvement in construction of pit latrines resulted in the support by the government for construction of latrines.

This project promoted the desirability and demand of household latrines and environmental cleanliness in the communities with government support. This also helped improve environmental and personal hygiene and hopefully reducing the prevalence of active trachoma. Of significance is that the exercise had opened up a channel of collaborative efforts in health matters between the community and local governments.

### Monitoring trachoma within health information system

Any control program will need a good monitoring system to supervise and administer. In Nigeria, trachoma, as many other blinding diseases, does not have a unified and standardized reporting system and a chain of transmission of information in the health information system. Although trachoma is listed in the HMIS diseases of the Ministry of Health, no concerted efforts have been made to document and report this neglected disease.

Trachoma control program or other programs [eg, Neglected Tropical Diseases (NTDs) control program] within which trachoma is domiciled, in the Ministry of Health, needs to strongly advocate for inclusion and enforcement of data collection on trachoma from all trachoma-endemic regions of the country. This is necessary for planning and monitoring of control programs.

### Health system and Government support

A sustainable and effective trachoma control requires a good health system with strong primary healthcare built upon an efficient multi-sectoral collaboration. However, many trachoma-endemic countries have a weak health system with even weaker primary healthcare. The health budget is often less than the UN recommended health budgetary allocation, and there are competing demands like HIV, malaria, tuberculosis, other sporadic infectious diseases like meningitis, gastroenteritis, including cholera. Most of the trachoma-endemic countries have the least health budget. Nigeria ranks 95^th^ country in budgetary allocation to health per person, of the 133 countries assessed in 1998, spending only $30 per capita (PPP).^30^

Endemic countries must make more budgetary allocation to health and strengthen their health system, especially primary healthcare, to curb not only trachoma but many other associated diseases. Stakeholders most advocate strongly for full integration of primary eye care into the primary health care and emphasize to governments that elimination of blinding trachoma in the short term could free up resources for other conditions and at the same time improve the standard of living for society’s most vulnerable populations.^22^

The setting up of trachoma control programs with the community-based mobilization and services in some states of Nigeria seems to have spurred the interest of local governments and other stakeholders with regard to eye care as wells as reactivated the dormant primary health centers in the states. In Sokoto state, these factors might have impressed the governments and played a significant role in encouraging the state government to set up a comprehensive eye care program. Other trachoma control states like Zamfara and Kebbi states may soon commence comprehensive eye care programs.

For its support of the global effort to eliminate blinding trachoma, the International Trachoma Initiative (ITI) builds coalitions with partners and agencies to implement the full SAFE strategy. The ITI works with Ministries of Health and other partners from basic planning and program development through implementation and evaluation. In less than 4 years, some of the world’s countries with the least resources have planned and implemented successful programs and demonstrated measurable short-term impact. These early successes point to the feasibility of implementing SAFE in a variety of program conditions, namely, geographic regions, population densities, and economic disparities.^22^ It, therefore, remains a challenge to the health administration and government of Nigeria to provide more budgetary allocation to the healthcare segment and also make use of these opportunities aiming for trachoma elimination by 2020, with the secondary aim of strengthening primary healthcare and general health systems.

## CONCLUSION

Unless more efforts are geared toward addressing various exceptional challenges facing local trachoma control programs, the hope of global elimination of one of the oldest causes of blindness by the year 2020 may prove a mirage. Various national and international trachoma control groups like GET 2020, NTD, need to keep up the momentum to sustain the achievements. The current momentum in reinvigorating primary healthcare with integration of eye care and health system strengthening provides opportunity to harness toward trachoma elimination.
